# Draft Genome Sequence of *Chryseobacterium* sp. Strain GSE06, a Biocontrol Endophytic Bacterium Isolated from Cucumber (*Cucumis sativus*)

**DOI:** 10.1128/genomeA.00577-16

**Published:** 2016-06-16

**Authors:** Jin-Ju Jeong, Byeong Hyeok Park, Hongjae Park, In-Geol Choi, Ki Deok Kim

**Affiliations:** a Laboratory of Plant Disease and Biocontrol, College of Life Sciences and Biotechnology, Korea University, Seoul, Republic of Korea; b Department of Biotechnology, Korea University, Seoul, Republic of Korea

## Abstract

*Chryseobacterium* sp. strain GSE06 is a biocontrol endophytic bacterium against the destructive soilborne oomycete *Phytophthora capsici,* which causes Phytophthora blight of pepper. Here, we present its draft genome sequence, which contains genes related to biocontrol traits, such as colonization, antimicrobial activity, plant growth promotion, and abiotic or biotic stress adaptation.

## GENOME ANNOUNCEMENT

The genus *Chryseobacterium* is a member of the family *Flavobacteriaceae* (1) and comprises species that are typically yellow rods with nonmotile non-spore-forming cells. *Chryseobacterium* species are abundant in water, soil, and chicken (2–4); they have also been isolated from plant rhizospheres (4–6), some of which have been shown to be involved in plant growth promotion and biocontrol of plant pathogens (7–9). *Chryseobacterium* sp. strain GSE06, examined in this study, exhibits biocontrol activity against a destructive soilborne oomycete, *Phytophthora capsici*, the causal agent of Phytophthora blight of pepper (10). The biocontrol activity of this strain might be related to its ability to colonize the rhizosphere of plant roots, compete with other microbes, or promote plant growth. Here, we report the draft genome sequence of the endophytic strain GSE06 with potential biocontrol traits, isolated from the surface-sterilized root of a cucumber plant grown in a field (Gunsan, South Korea) in 2002 (10).

Genome sequencing of strain GSE06 was performed using the Illumina MiSeq platform at the Computational and Synthetic Biology Laboratory, Korea University (Seoul, South Korea). A total of 2,065,808 paired-end reads (1,239.5 Mb, 232.6-fold coverage) were produced from paired-end sequencing of the genomic library, with an average insert size of 500 bp. Low-quality reads were trimmed, using a quality threshold of Q30. The trimmed reads were used for *de novo* assembly using the SPAdes assembler (11). The reads were assembled to 47 scaffolds with a total length of 5,329,438 bp and a G+C content of 36.1%. The maximum length and *N*_50_ value of the contigs were 1,437,779 bp and 611,595 bp, respectively. Genome annotation was conducted using the NCBI Prokaryotic Genome Annotation Pipeline (PGAP) service. In total, 4,622 coding sequences were predicted by PGAP; among these, 3,701 (80.1%) coding sequences exhibited sequence similarity to known genes in the NCBI database. In addition, 75 tRNAs and one 5S rRNA, one 16S rRNA, and one 23S rRNA sequence were retrieved.

The genome of strain GSE06 contains genes related to colonization ability (e.g., motility ability and biofilm formation), antimicrobial activity (e.g., enoyl-coenzyme A [CoA] hydratase, polyketide cyclase, and thiazole) (12, 13), and abiotic or biotic stress management (e.g., superoxide dismutase, hydrogen peroxidase, catalase, and peroxidase) (14). The genome analysis also revealed genes related to plant growth promotion, such as ammonia, siderophore, urease, indole-3-acetic acid (IAA), and phosphate solubilization (15). In conclusion, the genome analysis of *Chryseobacterium* sp*.* GSE06 will lead to an in-depth understanding of the biocontrol traits of the strain, such as colonization, antimicrobial activity, plant growth promotion, and environmental stress adaptation.

### Nucleotide sequence accession numbers.

This whole-genome shotgun project has been deposited in the DDBJ/EMBL/GenBank under the accession no. LUVZ00000000. The version described in this paper is the first version, LUVZ01000000.
